# Rapid speciation of mycobacteria with simultaneous detection of MDR tB

**DOI:** 10.1186/1471-2334-14-S3-P43

**Published:** 2014-05-27

**Authors:** Barnali Kakati, Rajiv Kumar Agarwal, Biswaroop Chatterjee, Aarti Kotwal, Pronoti Sarkar, Sudhir Singh, Victor Masih, Moirangthem Ajit Singh

**Affiliations:** 1 Department of Microbiology, Himalayan Institute of Medical Sciences, Dehradun-248140, India

## Background

Multidrug resistance (MDR) TB is on the rise globally and is associated with significant morbidity and mortality. Conventional culture based drug susceptibility testing (DST) for TB drugs is time consuming, leading to diagnostic delays contributing to higher TB morbidity and mortality and exacerbation of ongoing transmission. Moreover most of the NTM(Non tuberculous mycobacteria) that are resistant to common anti tB drugs, can be falsely labeled as MDR tB if not identified correctly. This study was aimed to identify *Mycobacterium* species and to determine the prevalence of multidrug resistant tuberculosis (MDR tB) by line probe assay.

## Methods

This study was done on 76 clinical isolates collected from TB patients who were on Anti tuberculous drug treatment since last six months but without any clinical improvement. Line probe assay was performed for detection, identification of species and drug susceptibility status.

## Results

Line probe assay detected 49 isolates (96%) of *M. tuberculosis*, 1 isolate (1.96%) of *M. avium* and *M. intracellulare* each. Out of 49 isolates of *M.tuberculosis* 9(18.4%) were Rifampicin resistant and 11(22.4%) INH resistant but what was alarming was that 9 isolates (18.4%) were MDR.

## Conclusion

The majority of multidrug resistant TB cases are due to ongoing transmission of multidrug resistant strains. This is most likely the result of diagnostic delay, thereby emphasizing the need for rapid diagnostics and line probe assay can be used for the rapid identification of *Mycobacterium* species and the determination of susceptibility to drugs, which may accelerate the time to effective MDR tB treatment.

